# B-Cell Depletion as Evidence for Shared Neuroimmune Pathways in Combined Central and Peripheral Demyelination: A Case Report and Literature Review

**DOI:** 10.3390/ijms27062810

**Published:** 2026-03-20

**Authors:** Laura-Elena Cucu, Alina Săcărescu, Cristina Grosu, Victor Constantinescu, Laura Cristina Baciu, Gabriela-Smărăndița Asaftei-Titianu, Cristina Gațcan, Costin Chirica, Otilia Elena Frăsinariu, Emilian Bogdan Ignat

**Affiliations:** 1 Doctoral School, Grigore T. Popa University of Medicine and Pharmacy, 700115 Iasi, Romania; dudau.laura-elena@d.umfiasi.ro (L.-E.C.);; 2 Clinical Rehabilitation Hospital, 700661 Iasi, Romania; 3 Department of Neurology, Grigore T. Popa University of Medicine and Pharmacy, 700115 Iasi, Romania; 4 Department of Mother and Child Medicine-Pediatrics, Grigore T. Popa University of Medicine and Pharmacy, 700115 Iasi, Romania

**Keywords:** combined central and peripheral demyelination, demyelination, neuroimmunology, chronic inflammatory demyelinating polyneuropathy, multiple sclerosis, B cells, anti-CD20 therapy

## Abstract

Combined central and peripheral demyelination (CCPD) is a rare neuroimmunological condition involving inflammatory demyelination of both the central nervous system (CNS) and peripheral nervous system (PNS). We report a chronic progressive CCPD case initially diagnosed as chronic inflammatory demyelinating polyneuropathy (CIDP) and treated with conventional CIDP-directed immunotherapies, with subsequent development of multiple sclerosis (MS)-like CNS demyelination. An extensive diagnostic evaluation excluded alternative infectious, metabolic, paraneoplastic, and antibody-mediated etiologies affecting either compartment. In the absence of a unifying pathogenic autoantibody, the combined clinical, radiological, cerebrospinal fluid, and electrophysiological findings support a shared immune-mediated process. Within this framework, B cells are implicated through antibody-independent mechanisms, including antigen presentation, pro-inflammatory cytokine production (e.g., IL-6), and amplification of Th1/Th17-driven inflammation. Interactions between B cells and the complement system via CR1 (CD35) and CR2 (CD21), together with dysfunction of the blood–brain barrier (BBB) and blood–nerve barrier (BNB), may facilitate parallel immune activation across both compartments. In this case, the observed radiological and electrophysiological stabilization under anti-CD20 therapy is consistent with a B-cell-driven pathogenic model in CCPD.

## 1. Introduction

Combined central and peripheral demyelination (CCPD) represents a rare neuroimmunological entity whose clinical and paraclinical features have been described in recent literature [1], yet therapeutic approaches remain poorly defined. Understanding CCPD through the lens of common immunopathological mechanisms rather than anatomical compartmentalization may provide insights into both pathogenesis and therapeutic strategies.

Despite their distinct anatomical localization, multiple sclerosis (MS) and chronic inflammatory demyelinating polyneuropathy (CIDP) share fundamental immunopathogenic mechanisms that suggest a common autoimmune pathogenesis. According to Melzer et al. 2014 [2], both conditions arise from a failure of immune tolerance followed by similar effector pathways of myelin damage. Demarcation between central and peripheral demyelinating disorders may be a conceptual convenience rather than a reflection of well-defined pathophysiological boundaries. Supporting this concept, previous studies have demonstrated subclinical central involvement in primarily peripheral demyelinating disease, including optic nerve changes in CIDP, evidenced by reduced retinal ganglion cell layer thickness [3], as well as biochemical and microstructural brain abnormalities on advanced neuroimaging [4]. Conversely, recent neurography studies have demonstrated peripheral nerve abnormalities in MS patients that appear independent of central nervous system (CNS) disease activity or treatment, supporting a shared inflammatory substrate across central and peripheral nervous systems [5].

The pathogenic mechanisms driving CCPD remain incompletely understood. To date, no common antibody targeting both the central and peripheral nervous systems has been identified in CCPD. While cases with fulminant presentation are more likely to reflect a shared pathogenic mechanism that remains to be elucidated, those with a chronic progressive course, such as ours, may represent either a unified demyelinating process affecting both compartments simultaneously, or an underlying autoimmune predisposition leading to sequential or overlapping manifestations of immune-mediated disease, similar to other neurological and rheumatological overlap syndromes [6]. Regardless of the specific antigenic triggers, or whether the process is unified or sequential, B lymphocytes appear to play a central pathogenic role in both compartments, making them attractive therapeutic targets.

B lymphocytes are essential mediators of adaptive immunity, primarily through antibody production and antigen presentation. CD20, a surface molecule expressed on all B cell developmental stages except plasma cells, serves as a key therapeutic target [7]. Beyond antibody secretion, B cells regulate immune responses through antigen presentation, cytokine production, and modulation of inflammatory pathways.

In the healthy CNS, B lymphocytes and plasma cells demonstrate limited parenchymal infiltration, residing primarily in perivascular spaces and meningeal compartments, particularly the dura mater [8]. Although B cells are present in normal CSF, plasma cells remain scarce, consistent with minimal baseline antibody production under homeostatic conditions [9].

Given the rarity of this condition and limited treatment data, reporting cases with positive therapeutic responses is essential for guiding clinical practice. To our knowledge, we present the first reported case from our country treated with ocrelizumab, adding to the limited literature on anti-CD20 therapy in CCPD, and demonstrating favorable clinical and paraclinical evolution. This case provides important evidence that anti-CD20 therapy can effectively address both central and peripheral demyelinating processes, potentially guiding treatment strategies for future CCPD cases. Additionally, we review the common pathogenic mechanisms between central and peripheral demyelination that provide the biological rationale for B cell-targeted therapy in CCPD.

## 2. Case Description

We present a 49-year-old man with a 12-year history of CIDP fulfilling the 2010 EFNS diagnostic criteria [10], with a chronic progressive sensorimotor course, treated with immunoglobulins, mycophenolate mofetil, and methylprednisolone. After 9 years of stability, he developed left retrobulbar optic neuritis. Visual Evoked Potentials (VEPs) showed increased P100 latency on the left, and optical coherence tomography (OCT) revealed retinal nerve fiber layer (RNFL) thinning, both suggesting optic nerve demyelination. Brain MRI revealed multiple bilateral periventricular demyelinating lesions. CSF analysis demonstrated elevated protein (45.4 mg/dL) and three oligoclonal bands exclusive to CSF. Extensive workup excluded alternative etiologies: p-ANCA, c-ANCA, ANA IgG panel, serum protein electrophoresis, vitamin B12, HIV, syphilis, hepatitis B/C, antineuronal antibodies (contactin-1, neurofascin-155, neurofascin-186, CASPR-1), TTR gene sequencing for hereditary transthyretin amyloidosis and AQP4 antibodies were all negative. Borrelia serology showed positive IgM with negative IgG, prompting treatment for borreliosis. MOG antibodies were detected at a low titer (1:20).

Over time, paraclinical investigations demonstrated bilateral optic nerve involvement, with more pronounced right-sided abnormalities. Serial brain and cervical MRI over two years revealed new demyelinating lesions in the left frontal subcortical region, left cerebellum, left mesencephalon, and cervical spinal cord, fulfilling the 2017 McDonald criteria for dissemination in time and space. Follow-up EMG demonstrated progressive deterioration in nerve conduction despite immunosuppression. Repeat CSF analysis showed a persistent single oligoclonal band with negative MOG and Borrelia antibodies.

Neurological clinical examination revealed bilateral steppage gait with foot drop and tetraparetic motor deficit, with distal predominance. Upper limbs showed weakness limited to ulnar grip, while lower extremity examination demonstrated proximal weakness (MRC 4/5) and more severe distal weakness (MRC 3/5), particularly affecting foot dorsiflexion, with mild left-sided predominance. Deep tendon reflexes were present at all levels, brisker on the right. Babinski sign was positive on the right and equivocal on the left. Generalized muscle atrophy was present in both upper and lower limbs, with trophic changes including hammer toes and bilateral pes cavus. Fasciculations, both spontaneous and provoked, were observed in the thighs. Sensory examination showed distal paresthesia in both lower extremities, with hypoesthesia to tactile, vibratory, and proprioceptive stimuli. Fine postural tremor was noted in the upper extremities.

Overall, the neurological examination demonstrated a mixed central and peripheral pattern, with predominant peripheral motor and sensory involvement associated with superimposed pyramidal signs.

**Figure 1:**
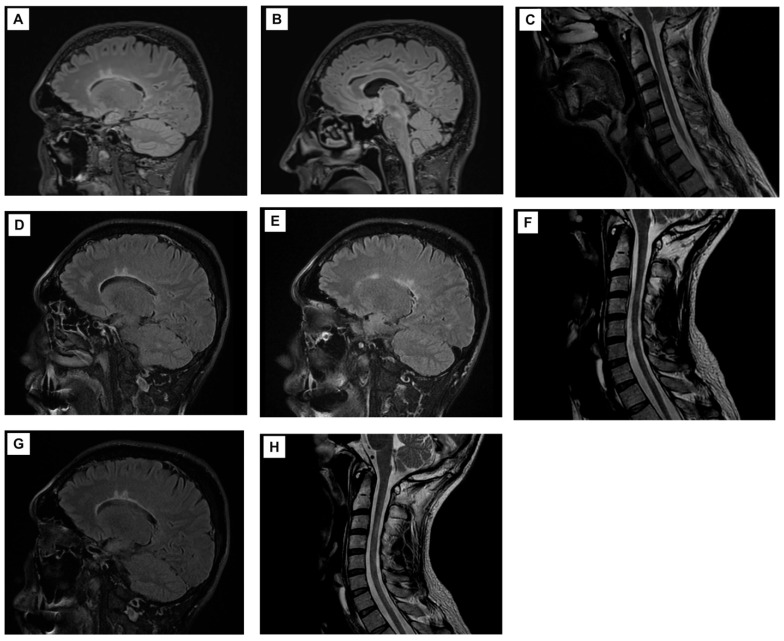
Serial Sagittal MRI. (**A**) 2022—Brain FLAIR: Hyperintense demyelinating lesions perpendicular to the lateral ventricles. (**B**) 2023—Brain FLAIR: Persistent periventricular demyelinating lesions and new cerebellar lesion. (**C**) 2023—Cervical Spine T2: No demyelinating lesions identified. (**D**,**E**) 2024—Brain FLAIR: Stable periventricular and cerebellar lesions with new subcortical demyelinating lesion. (**F**) 2024—Cervical Spine T2 frFSE: Hyperintense lesions at C3–C4 and C5–C6 levels. (**G**) 2025—Brain FLAIR: Stable appearance of periventricular, cerebellar, and subcortical lesions. (**H**) 2025—Cervical Spine T2 frFSE: Stable cervical cord lesions with no new lesions detected.

Given confirmed concurrent CNS and PNS demyelination, Ocrelizumab was initiated. After one year of treatment, a comprehensive reassessment was performed. Brain and spinal MRI (Figure 1) showed stable disease with no new lesions. Nerve conduction studies (Table 1) demonstrated improvement in nerve conduction velocities and latencies, suggesting stabilization of the demyelinating process. Ophthalmological evaluation revealed progression to bilateral optic neuritis, predominantly affecting the right eye. VEPs showed marked P100 delay and reduced amplitude bilaterally (worse on the right), while OCT confirmed progressive RNFL and GCL thinning, more accentuated in the right eye. Notably, the initial ophthalmological assessment (showing unilateral left optic nerve involvement) had been performed before the bilateral optic neuritis relapse that occurred immediately prior to Ocrelizumab initiation, precluding direct comparison. The absence of new CNS or PNS lesions on MRI and improvement in nerve conduction studies suggest successful disease control. The ophthalmological findings likely represent structural injury from the pre-treatment bilateral relapse rather than ongoing inflammatory activity under B-cell depletion.

## 3. Discussion

### 3.1. Diagnostic Evaluation

In the present case, an extensive diagnostic evaluation was undertaken to exclude alternative causes of demyelination involving the CNS (Table 2), or the PNS (Table 3). A wide range of differential diagnoses was systematically considered, including hereditary neuropathies, infectious, paraneoplastic, vasculitic, metabolic, and granulomatous disorders, as well as other inflammatory demyelinating diseases. These alternatives were excluded based on clinical features, electrophysiological findings, CSF analysis, neuroimaging characteristics, and disease evolution, as summarized in the differential tables. The findings fulfilled established diagnostic criteria for CIDP and MS, respectively.

To contextualize the simultaneous central and peripheral involvement in our patient, we systematically evaluated conditions that can produce combined demyelination affecting both the CNS and PNS (Table 4).

Regarding infectious triggers, EBV testing has a very low diagnostic yield in chronic demyelinating conditions. EBV contributes to MS susceptibility at a population level but does not provide diagnostic or therapeutic information in established MS [27], and EBV neurological disease is typically acute, systemic, and CSF-inflammatory rather than chronically progressive [28]. Accordingly, current CCPD literature does not recommend EBV testing unless there is clinical suspicion of acute viral involvement [1], and such testing was not performed in our patient.

CCPD, a term first introduced by Amit in 1992 [32], refers to demyelinating lesions affecting both the central and peripheral nervous systems. It is increasingly recognized not as a single disease entity, but rather as a heterogeneous group of disorders, as some cases demonstrate a fulminant course while others, such as the present case, follow a chronic progressive evolution [1,31]. To contextualize the simultaneous central and peripheral involvement in our patient, we first examine the distinct pathogenic pathways of CIDP and MS and their potential points of convergence.

This case is notable for disease progression in both central (new MRI lesions) and peripheral (worsening conduction velocities) compartments despite conventional immunosuppressive treatment with Mycophenolate Mofetil and corticosteroids, demonstrating an aggressive disease phenotype with parallel activity across nervous system compartments. However, transition to B-cell depletion therapy with Ocrelizumab achieved successful disease control, with stabilization of MRI findings and improvement in nerve conduction studies after one year of treatment.

Although coexistence of MS and CIDP has been reported, the temporal evolution, progressive course, and parallel disease activity affecting both compartments support a unifying diagnosis within the CCPD spectrum rather than a coincidental association of two unrelated disorders.

### 3.2. Pathogenic Framework

**Figure 2:**
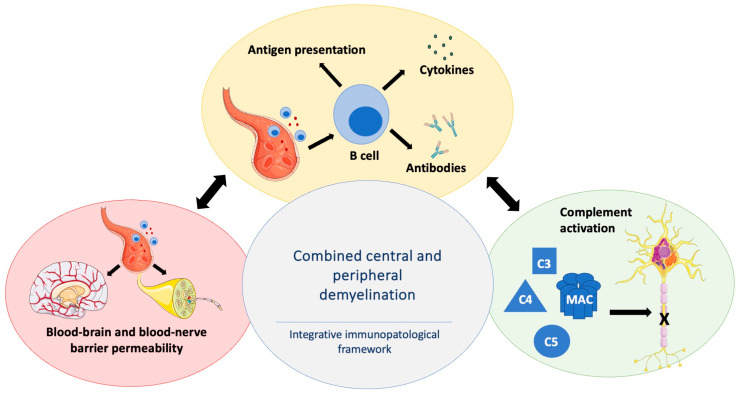
Integrative immunopathogenic model of combined central and peripheral demyelination (CCPD). Barrier dysfunction at the blood–brain and blood–nerve interfaces permits immune cell entry and diffusion of soluble inflammatory mediators. B cells contribute to disease through antigen presentation, cytokine secretion, and antibody-dependent or antibody-independent mechanisms. Complement activation acts as an interconnected effector pathway, amplifying inflammation and tissue injury, including formation of the membrane attack complex (MAC), thereby sustaining demyelination across both compartments. This figure includes elements adapted from Servier Medical Art (https://smart.servier.com), licensed under CC BY 4.0.

Current evidence suggests that combined central and peripheral demyelination may be conceptualized through several interconnected immunopathological processes rather than a single disease-specific mechanism (Figure 2). First, barrier dysfunction may act as a permissive step, facilitating immune cell trafficking across the blood–brain and blood–nerve interfaces [33]. Second, B-cell–mediated immunity appears to play an important contributory role through antibody production, antigen presentation, and cytokine secretion [7], reflecting compartment-specific immune responses in which analogous mechanisms may drive distinct inflammatory patterns in the central and peripheral nervous systems [34,35]. Third, complement activation may contribute to tissue injury by amplifying inflammatory cascades and modulating B-cell function [36]. Given the rarity of CCPD and the absence of disease-specific mechanistic studies, evidence supporting these processes is primarily derived from multiple sclerosis and chronic inflammatory demyelinating polyneuropathy, which serve as the most extensively characterized reference models of immune-mediated demyelination in the central and peripheral nervous systems, respectively. These components are therefore discussed below as an integrative framework to contextualize the observed combined phenotype.

### 3.3. Barrier Dysfunction

The 12-year interval between PNS and CNS demyelination onset may reflect the inherently greater permeability of the BNB (Blood-Nerve Barrier) compared to the BBB (Blood–Brain Barrier). Endoneurial capillaries exhibit fewer tight junction apposition sites than cerebral microvessels [37,38], potentially lowering the threshold for immune-mediated peripheral demyelination in individuals with predisposition. However, what constitutes this predisposition remains unclear, as no consistent genetic, immunological, or environmental factor has been identified across reported CCPD cases. A shared genetic predisposition was also considered during the diagnostic evaluation. The HLA-DRB1*15:01 allele, which is strongly associated with multiple sclerosis in the Romanian population [39], has also been linked to anti-neurofascin-155 antibody–positive CIDP [40]. However, our patient tested negative for anti-NF155 antibodies.

### 3.4. B-Cell Mediated Immunity

**Figure 3:**
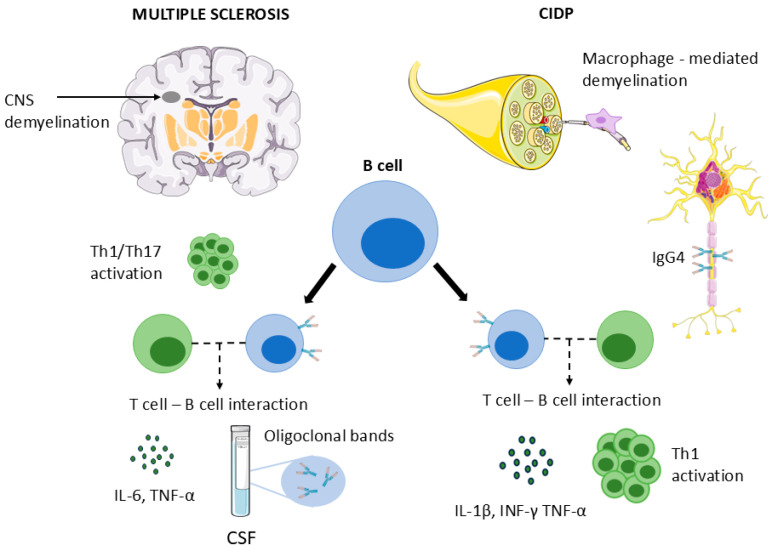
Central role of B cells in the pathogenesis of combined central and peripheral demyelination. Schematic illustration of the central role of B cells in combined central and peripheral demyelination. In MS, B cells are proposed to contribute to CNS demyelination through antigen presentation, cytokine secretion, and intrathecal antibody production, promoting Th1 and Th17 responses. In CIDP, B cells are proposed to contribute to macrophage-mediated demyelination through cytokine-dependent and antibody-independent mechanisms. Targeting CD20^+^ B cells represents a shared therapeutic strategy capable of modulating inflammatory activity in both compartments. This figure includes elements adapted from Servier Medical Art (https://smart.servier.com), licensed under CC BY 4.0.

From a pathogenetic perspective, CIDP has traditionally been viewed as a disorder driven primarily by macrophage-mediated demyelination, supported by Th1-type immune responses and breakdown of the blood–nerve barrier. Albuminocytologic dissociation, as observed in our patient, reflects increased permeability of this barrier rather than intrathecal immunoglobulin synthesis. However, accumulating evidence indicates that humoral immunity and B cells also contribute to CIDP pathogenesis. Pathogenic IgG4 autoantibodies against nodal and paranodal proteins define specific CIDP subtypes, and even in antibody-negative CIDP, B-cell involvement has been suggested through altered B-cell subsets, cytokine production, and clinical responses to B-cell–depleting therapies [34,41,42]. Clinical improvement following rituximab has been reported even in seronegative CIDP, supporting antibody-independent roles for B cells in disease propagation [43] (Figure 3).

In MS, B cells are now recognized as central contributors to disease pathogenesis. Beyond antibody production, B cells act as antigen-presenting cells, regulate T-cell activation, and secrete pro-inflammatory cytokines, including IL-6 and TNF-α, thereby promoting pathogenic Th1 and Th17 responses [44]. The presence of oligoclonal bands in CSF and the identification of B cells within demyelinating CNS lesions underscore their pathogenic relevance. The robust clinical efficacy of anti-CD20 therapies, including ocrelizumab, further supports the pivotal role of B cells in MS [35,45] (Figure 3).

### 3.5. Complement Activation

**Figure 4:**
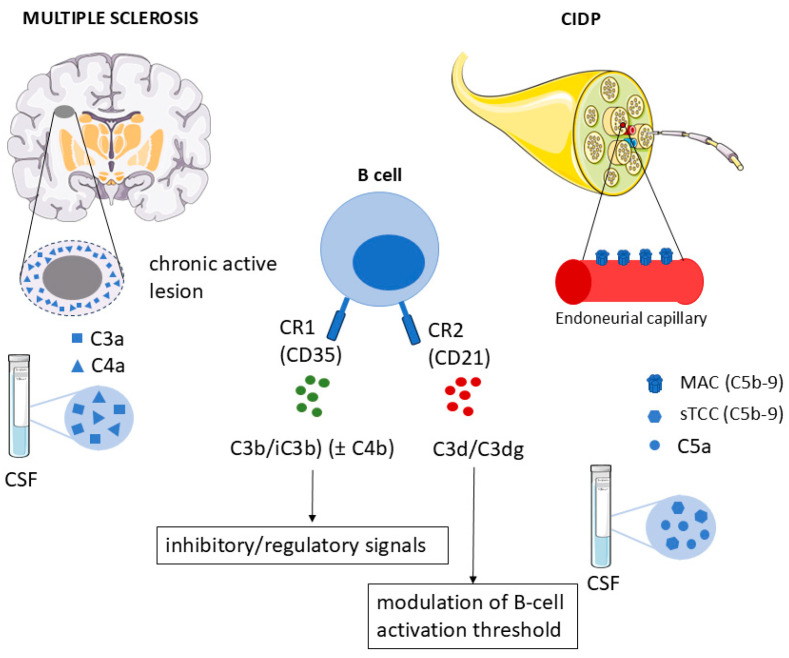
Complement activation and its interaction with B cells in combined central and peripheral demyelination. Schematic representation of complement activation as a shared pathogenic mechanism in CIDP and multiple sclerosis (MS). In CIDP, activation of the terminal complement pathway leads to generation of C5a and soluble terminal complement complex (sTCC), with membrane attack complex (C5b-9) deposition predominantly on endoneurial capillaries, contributing to blood–nerve barrier disruption and peripheral nerve injury. In MS, complement activation is reflected by elevated CSF levels of C3a and C4a and by complement deposition at the rims of chronic active CNS lesions, particularly in progressive disease. Complement fragments such as C3b, iC3b, and C3d interact with complement receptors CR1 (CD35) and CR2 (CD21) expressed on B cells, modulating B-cell activation thresholds, cytokine production, and immune responses in a context-dependent manner. This representation highlights complement activation as a mechanistic link between humoral immunity, B-cell function, and tissue injury across central and peripheral compartments. This figure includes elements adapted from Servier Medical Art (https://smart.servier.com), licensed under CC BY 4.0.

Complement activation provides an additional mechanistic link between humoral immunity and tissue injury in both central and peripheral demyelination. In CIDP, elevated serum and CSF levels of C5a and soluble terminal complement complex correlate with disease severity, and nerve biopsy studies demonstrate membrane attack complex deposition on endoneurial capillaries, consistent with targeted immune-mediated complement activation rather than passive protein leakage [46,47]. In MS, elevated CSF levels of C3a and C4a correlate with clinical severity and axonal injury, and complement deposition is particularly prominent in progressive disease and at the rims of chronic active lesions [48,49,50] (Figure 4).

Complement activation is tightly linked to B-cell function. Complement fragments such as C3b, iC3b, and C3d bind antigens and immune complexes and interact with complement receptors expressed on B cells, including CR1 (CD35) and CR2 (CD21). Engagement of these receptors modulates B-cell activation thresholds, proliferation, antibody production, and memory responses, exerting both activating and inhibitory effects depending on context [36,51,52,53]. Dysregulation of complement receptor signaling has been implicated in autoimmunity through altered B-cell tolerance and activation thresholds [54] (Figure 4).

Bonin et al. analyzed cerebrospinal fluid from MS and CIDP patients and demonstrated disease-specific cytokine profiles: SCGF-β and SDF-1α were elevated in CIDP, whereas IL-12 predominated in MS. These findings support the concept that central and peripheral demyelination exhibit distinct inflammatory patterns despite analogous pathogenic mechanisms, reflecting compartment-specific immunopathogenesis [55].

### 3.6. Therapeutic Implications

While anti-CD20 therapy is well established in multiple sclerosis, its role in CIDP remains investigational. Certain CIDP subtypes are associated with pathogenic IgG4 autoantibodies directed against nodal and paranodal antigens, including neurofascin-155 (NF155), contactin-1 (CNTN1), and contactin-associated protein 1 (CASPR1). These antibodies, generated by B-cell lineages originating from CD20^+^ precursors, disrupt axo–glial junctions, impair saltatory conduction, and lead to a distinctive phenotype characterized by poor response to conventional therapies such as intravenous immunoglobulins and corticosteroids [34]. In contrast, these patients frequently demonstrate clinical improvement following B-cell–depleting therapy with rituximab, which targets upstream B-cell populations involved in pathogenic antibody generation [56].

Clinical evidence supporting anti-CD20 therapy in CIDP is accumulating. A systematic review and meta-analysis including 96 CIDP patients from 15 studies reported an overall response rate of 75% to rituximab, with particularly robust responses observed in patients harboring IgG4 antibodies against nodal or paranodal antigens, although outcome assessment was largely based on clinical measures [42]. In a retrospective cohort of 11 patients with refractory CIDP, rituximab treatment resulted in significant clinical improvement within 2–3 months, despite only one patient testing positive for anti-neurofascin antibodies, suggesting that therapeutic benefit may extend beyond antibody-positive subgroups [43].

Importantly, efficacy of B-cell–depleting therapy has also been reported in CCPD, with successful outcomes documented in three cases treated with rituximab [57,58,59] and one case treated with ocrelizumab [60]. While rituximab remains the most widely used anti-CD20 agent in peripheral demyelinating disorders, ocrelizumab offers theoretical advantages in CCPD due to its proven efficacy in the central compartment [35,45] and emerging evidence of benefit in CIDP [60], making it a rational choice in CCPD.

In this context, biomarkers reflecting neuroaxonal injury may provide additional insight into treatment response to B-cell–depleting therapies. Neurofilament light chain (NfL) is a nonspecific marker of neuronal damage, useful across a wide range of neurological conditions, reflecting axonal injury regardless of its underlying cause [61]. In MS, it serves as a prognostic and therapy-monitoring biomarker, with elevated levels correlating with disease severity, progression, and treatment response [61,62]. In CIDP, though less extensively studied, it represents a promising indicator of disease activity, with elevated levels in early-stage disease signaling severe inflammatory-mediated axonal damage and poorer outcomes [61,63]. Although NfL levels were not measured in our patient, future studies incorporating longitudinal biomarker assessment may help clarify its role in monitoring disease activity in CCPD.

Beyond antibody depletion, anti-CD20 therapy exerts broader immunomodulatory effects on T-cell–mediated inflammation. B cells function as antigen-presenting cells and produce pro-inflammatory cytokines, particularly interleukin-6, which is critical for Th17 differentiation [64]. Depletion of CD20^+^ B cells reduces T-cell activation and dampens Th1/Th17-driven inflammatory cascades [65]. This mechanism is particularly relevant in MS, where Th1 and Th17 responses predominate, and in typical CIDP without identifiable pathogenic antibodies, where macrophage-mediated demyelination is driven by Th1-type immune responses [41]. Thus, anti-CD20 therapy targets shared immunopathological pathways across central and peripheral demyelinating disorders, even in the absence of detectable pathogenic antibodies.

### 3.7. Limitations and Future Directions

Important limitations in CCPD management include the unknown antigenic targets (whether shared or distinct autoantibodies drive CNS and PNS demyelination), the poorly characterized natural history of the disease, and the undefined optimal duration of B cell-depleting therapy. The absence of standardized diagnostic criteria further complicates systematic study of this rare condition. Given that randomized controlled trials are unfeasible due to disease rarity, detailed case reports or case series with long-term follow-up remain essential for advancing therapeutic knowledge.

These limitations further suggest that CCPD may be underrecognized in clinical practice. In patients with multiple sclerosis, sensory disturbances or motor deficits are frequently interpreted solely within the context of central nervous system pathology, potentially overlooking concomitant peripheral nerve involvement that may only be identifiable through targeted electrophysiological evaluation. Conversely, in patients with CIDP, central nervous system involvement may remain clinically silent or manifest with subtle features that are overshadowed by prominent peripheral deficits, particularly given that brain MRI is not routinely performed in this population. As a result, combined central and peripheral demyelination may remain undetected, contributing to diagnostic delay and incomplete therapeutic response.

**Figure 5:**
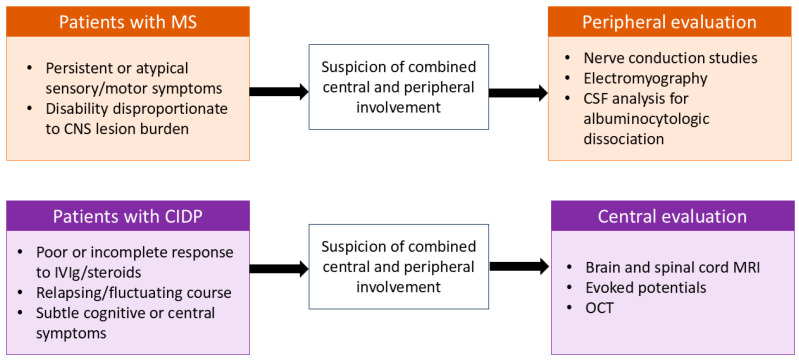
Conceptual framework for the recognition and targeted evaluation of combined central and peripheral demyelination. Schematic illustration highlighting clinical scenarios in which combined central and peripheral nervous system involvement could be considered.

These observations underscore the need for a more systematic approach to identifying patients at risk for CCPD. Clinical scenarios that may warrant evaluation of the complementary nervous system compartment include atypical or relapsing disease courses, disproportionate disability relative to objective findings, and inadequate response to standard immunotherapies. In such cases, structured history taking focused on central or peripheral relapse patterns, electrophysiological assessment, evoked potentials, and targeted neuroimaging may improve detection of combined involvement (Figure 5). Establishing evidence-based screening strategies in selected patient subgroups represents an important step toward refining diagnostic criteria and optimizing individualized treatment approaches for CCPD.

## 4. Conclusions

CCPD pathogenesis can be conceptualized through three interconnected mechanisms: barrier dysfunction, B-cell immunity, and complement activation that converge to produce demyelination across both CNS and PNS compartments. The clinical and electrophysiological stabilization following ocrelizumab provides evidence for the central role of B cells in driving disease across both compartments, independent of detectable pathogenic antibodies.

These findings support anti-CD20 therapy as a rational approach in CCPD. As standardized diagnostic criteria and treatment protocols remain undefined, detailed case reports with long-term follow-up are essential for advancing our understanding and management of this rare condition.

## Figures and Tables

**Table 1:** Nerve conduction studies at CIDP diagnosis (2014), pre-Ocrelizumab baseline (2024), and one-year follow-up (2025).

	Nerve		2014	2024	2025	Evolution
Motor	Ulnaris L (elbow)	Latency (ms)	11.5	11.2	9.6	↓
		Amplitude (mV)	10.04	6.7	4.7	↓
		Conduction velocity (m/s)	30.8	34.8	37.0	↑
	Ulnaris L (arm)	Latency (ms)	-	16.8	13.3	↓
		Amplitude (mV)	-	6.0	4.2	↓
		Conduction velocity (m/s)	-	23.4	29.4	↑
	Medianus L (elbow)	Latency (ms)	12.5	12.3	11.1	↓
		Amplitude (mV)	4.48	5.6	4.9	↓
		Conduction velocity (m/s)	34.8	36.5	36.5	=
Sensory	Radialis R	Latency (ms)	-	3.7	2.9	↓
		Amplitude (mV)	-	10.6	14.2	↑
		Conduction velocity (m/s)	-	39.9	44.1	↑
	Medianus L	Latency (ms)	4.9	7.1	5.1	↓
		Amplitude (mV)	5.6	12.1	6.3	↓
		Conduction velocity (m/s)	32.7	30.3	44.4	↓
	Suralis L	Latency (ms)	-	4.4	4.4	=
		Amplitude (mV)	-	9.6	9.5	=
		Conduction velocity (m/s)	-	33.0	31.8	↓

↑ improvement compared to previous examination, ↓ deterioration compared to previous examination, = no significant change between evaluations.

**Table 2:** Differential diagnosis in CNS demyelination.

Differential	CNS Features Supporting	CNS Features Arguing Against	Conclusion
Adult-onset leukodystrophy	Can produce extensive white-matter changes and occasionally peripheral involvement [11].	MS-typical MRI (periventricular, juxtacortical, infratentorial), no early cognitive/systemic signs of leukodystrophy.	Unlikely. Pattern favours acquired demyelination.
Mitochondrial CNS disease	Mitochondrial disorders may involve CNS (stroke-like episodes, ataxia, epilepsy) [12].	No stroke-like events, no basal ganglia lesions, no lactic acidosis or multisystem disease; MRI lesions are demyelinating, not metabolic.	Excluded. Does not match phenotype.
AQP4–NMOSD	Optic neuritis and myelitis are core NMOSD features [13]	AQP4-IgG negative; no LETM, no area postrema syndrome; MRI pattern typical for MS, not NMOSD.	Excluded. Criteria not met.
MOGAD	MOGAD can cause optic neuritis and multifocal demyelination [14,15].	Only low-titer, transient MOG-IgG; MRI lacks fluffy/ADEM-like lesions; no recurrent steroid-responsive optic neuritis.	Excluded. Features are more consistent with MS.
Multiple sclerosis	Typical MRI distribution, optic neuritis, dissemination in time and space, and CSF OCBs strongly support MS [16].	None significant; alternative diagnoses do not better explain the CNS findings.	Supported. CNS disease fulfills criteria for MS.

**Table 3:** Differential diagnosis of chronic peripheral neuropathies.

Differential	PNS Features Supporting	PNS Features Arguing Against	Conclusion
Amyloid neuropathy	Chronic neuropathy possible in hereditary or acquired amyloidosis [17]	Typically axonal, often painful with autonomic failure; does not cause primary demyelination with albuminocytologic dissociation [17].	Excluded. Phenotype incompatible.
Paraneoplastic neuropathy	Can present as sensory or sensorimotor neuropathy, occasionally demyelinating [18,19].	No malignancy over >10 yrs; course and NCS fit idiopathic CIDP, not paraneoplastic patterns.	Excluded. No tumour or serological support.
Hereditary neuropathy (CMT)	Advanced CMT can mimic chronic demyelinating neuropathy [20,21]. The patient has atrophic changes that could fit CMT	Onset at 37 years is rarer. Early sural sparing and patchy NCS contradict the uniform slowing typical of CMT. Very high CSF protein and no family history also argue against heredity; atrophy reflects chronic CIDP, not specific to CMT.	Excluded. Findings favor acquired CIDP.
Paranodal antibody neuropathy (NF155, CNTN1, CASPR1, NF186)	Severe demyelination with poor IVIG response can resemble paranodal disease [22,23].	All antibodies negative; lacks typical features (distal tremor, ataxia); NCS evolution consistent with conventional CIDP.	Excluded subtype. Fits seronegative CIDP.
CIDP	Typical CIDP pattern with adult onset, chronic progression, early sural sparing, demyelinating NCS abnormalities, and marked albuminocytologic dissociation [10,24].	Partial IVIG response, but common in CIDP (up to one-third nonresponders).	Supported. Best explanation for PNS findings.

**Table 4:** Differential diagnosis of CCPD.

Differential	Features Supporting Combined Involvement	Features Arguing Against	Conclusion
Lyme neuroborreliosis	Lyme can affect CNS and PNS via meningitis, radiculoneuritis, or neuropathy [25,26]	Initial tests negative. No CSF pleocytosis, no painful radiculitis, no difference in the clinical picture after antibiotics	Excluded. Clinical and CSF profile incompatible.
EBV-related demyelination (background consideration)	EBV is associated with MS susceptibility; rare cases show acute CNS/PNS involvement [27,28].	EBV neurological disease is acute, systemic, and inflammatory with CSF pleocytosis. Neuropathy is usually GBS/AIDP-like, not CIDP.	Not a clinical differential. Background risk factor only.
Systemic vasculitis	Vasculitis may cause CNS lesions and PNS neuropathy [29].	Typically causes painful, asymmetric axonal neuropathy (mononeuritis multiplex) and ischemic CNS lesions, absent in our case; no systemic features.	Excluded. Phenotype incompatible.
Sarcoidosis (neurosarcoidosis + neuropathy)	Sarcoidosis can produce combined CNS involvement and peripheral neuropathy [30]	No systemic sarcoidosis; MRI lacks leptomeningeal/nodular enhancement; neuropathy is demyelinating, not a typical sarcoid pattern.	Excluded. No supportive systemic or imaging features.
MS + CIDP (overlap of two autoimmune demyelinating disorders)	CNS findings fulfill MS criteria; PNS findings fulfill CIDP criteria; coexistence is recognised in practice and literature [10,16]	Temporal and immunological patterns suggest linked rather than independent diseases; the combined phenotype better fits the CCPD-spectrum patterns.	Clinically plausible, yet it offers a less integrated explanation than CCPD
CCPD	Unified CNS + PNS demyelination with MS-like lesions and CIDP-like neuropathy matches CCPD descriptions, which are often antibody-negative and show mixed MS/CIDP features [1,31]. Shared partial response to B-cell therapy supports a systemic immune process.	No known paranodal antibodies, but many CCPD cases are seronegative.	Favoured diagnosis. Best explains the combined demyelinating phenotype.

## Data Availability

The data presented in this study are available on request from the corresponding author due to the privacy of the data.

## Abbreviations

The following abbreviations are used in this manuscript:

ANA	Antinuclear antibodies
ANCA	Anti-neutrophil cytoplasmic antibodies
AQP4	Aquaporin-4
BBB	Blood–brain barrier
BNB	Blood-nerve barrier
CASPR1	Contactin-associated protein 1
CCPD	Combined central and peripheral demyelination
CD20/CD21/CD35	Cluster of differentiation 20/21/35
CIDP	Chronic inflammatory demyelinating polyneuropathy
CMT	Charcot-Marie-Tooth disease
CNTN1	Contactin-1
CNS	Central nervous system
CSF	Cerebrospinal fluid
EBV	Epstein–Barr virus
EFNS	European federation of neurological societies
EMG	Electromyography
FLAIR	Fluid-attenuated inversion recovery
HIV	Human immunodeficiency virus
IgG	Immunoglobulin G
MAC	Membrane attack complex
MOG	Myelin oligodendrocyte glycoprotein
MRI	Magnetic resonance imaging
MS	Multiple sclerosis
NCS	Nerve conduction studies
NF155	Neurofascin-155
NfL	Neurofilament light chain
NMO	Neuromyelitis optica
NMOSD	Neuromyelitis optica spectrum disorder
OCT	Optical coherence tomography
PNS	Peripheral nervous system
RNFL	Retinal nerve fiber layer
T2	T2-weighted imaging
VEP	Visual evoked potentials
